# The Interplay between ROS and Ras GTPases: Physiological and Pathological Implications

**DOI:** 10.1155/2012/365769

**Published:** 2011-11-30

**Authors:** Elisa Ferro, Luca Goitre, Saverio Francesco Retta, Lorenza Trabalzini

**Affiliations:** ^1^Department of Biotechnology, University of Siena, Via Fiorentina 1, 53100 Siena, Italy; ^2^Department of Clinical and Biological Sciences, University of Torino, Regione Gonzole 10, 10043 Orbassano, Italy

## Abstract

The members of the RasGTPase superfamily are involved in various signaling networks responsible for fundamental cellular processes. Their activity is determined by their guanine nucleotide-bound state. Recent evidence indicates that some of these proteins may be regulated by redox agents. Reactive oxygen species (ROSs) and reactive nitrogen species (RNSs) have been historically considered pathological agents which can react with and damage many biological macromolecules including DNA, proteins, and lipids. However, a growing number of reports have suggested that the intracellular production of ROS is tightly regulated and that these redox agents serve as signaling molecules being involved in a variety of cell signaling pathways. Numerous observations have suggested that some Ras GTPases appear to regulate ROS production and that oxidants function as effector molecules for the small GTPases, thus contributing to their overall biological function. Thus, redox agents may act both as upstream regulators and as downstream effectors of Ras GTPases. Here we discuss current understanding concerning mechanisms and physiopathological implications of the interplay between GTPases and redox agents.

## 1. Introduction

The Ras GTPase superfamily includes low molecular weight GTP-binding and hydrolyzing (GTPases) proteins that act as molecular switches by coupling extracellular signals to different cellular responses, thus controlling cellular signaling pathways responsible for growth, migration, adhesion, cytoskeletal integrity, survival, and differentiation. The three human Ras proteins, H-Ras, N-Ras, and K-Ras, are the founding members of this large superfamily of small GTPases comprising over 150 human members with evolutionarily conserved orthologs found in *Drosophila, C. elegans, S. cerevisiae, S. pombe, Dictyostelium,* and plants. This superfamily is divided into families and subfamilies on the basis of sequence and functional similarities (Table 1). The five major families are Ras, Rho, Rab, Arf, and Ran [1]. In addition to the different Ras isoforms, the Ras family includes Rap, R-Ras, Ral, and Rheb proteins, also regulating signaling networks. Rho GTPase family includes the well-characterized family members Rac1, RhoA, and Cdc42, each of which is associated with unique phenotypes and functions [2–4]. Rab proteins comprise the largest branch of superfamily and regulate intracellular vesicular transport and trafficking of proteins. Like the Rab proteins, Arf family proteins are involved in regulation of vesicular transport. The Ran protein is the most abundant small GTPase in the cell and is best known for its function in nucleocytoplasmic transport of both RNA and proteins [1].

Although being similar to the heterotrimeric G protein *α* subunit in biochemical mechanism and function, Ras GTPases function as monomeric GTP-binding proteins. The functional diversity of these proteins is based on variations in structure, posttranslational modifications that dictate specific subcellular localizations, and proteins that act as regulators and effectors [1, 5].

Signal transduction through Ras proteins occurs by reversible binding of GTP, while the inactive form is bound to GDP. Switching between these two states is regulated by three distinct types of protein modulator agents: Guanine nucleotide Exchange Factors (GEFs) catalyze the exchange of GDP with GTP to promote Ras activation, whereas GTPase-Activating Proteins (GAPs) deactivate the Ras protein by stimulating hydrolysis of bound GTP to GDP. Deactivation can also be achieved by association with Guanine Nucleotide Dissociation Inhibitors (GDIs), which prevent membrane association, and GDP dissociation. All of these regulatory proteins are themselves affected by diverse upstream signals, which serve to activate or inactivate Ras GTPase signaling pathways. The transition of Ras proteins between the GDP- and GTP-bound states is accompanied by a conformational change that greatly enhances their affinity for downstream effectors [6]. The interaction between the active GTP-bound GTPase and the effector molecule leads to activation of downstream signal transduction pathways.

In addition to these protein regulatory factors, many of the Ras superfamily small GTPases have been shown to be redox sensitive, and their known conserved redox-sensitive sequences have been termed the NKCD, GXXXXGK(S/T)C, and CGNKXD motifs. The action of redox agents on these redox-sensitive GTPases is similar to that of guanine nucleotide exchange factors in that they perturb GTPase nucleotide-binding interactions that result in the enhancement of the guanine nucleotide exchange of small GTPases [7]. 

For many years, the generation of intracellular redox agents such as reactive oxygen species (ROS) and reactive nitrogen species (RNS) was viewed solely as the unregulated by-product of aerobic metabolism and other enzymatic processes, and redox agents have been historically considered pathological agents which can react with and damage many biological macromolecules including DNA, proteins, and lipids. However, over the last years a growing number of reports have suggested that mammalian cells can rapidly respond to ligand stimulation with a change in intracellular ROS thus indicating that the production of intracellular ROS is tightly regulated and that these redox agents serve as intracellular signaling molecules being involved in a variety of cell signaling pathways, including growth factor signaling [8, 9], inflammation [10], engagement of integrins [11, 12], and adhesion to extracellular matrix [13]. The precise means of regulation is not completely understood. However, numerous observations have suggested that the Ras GTPases appear to regulate ROS production and that oxidants function as effector molecules for the small GTPases, thus contributing to their overall biological function [14].

Here we discuss current understanding concerning the interplay between GTPases and redox agents. The discussion also takes into account pathological implications of alterations of both ROS regulation by small GTPases and small GTPases regulation by ROS.

## 2. ROS Regulation by RasGTPases

Among the major source of ROS, NADPH oxidases have been demonstrated to play a fundamental role in the compartmentalization of ROS production and redox signaling [15]. Besides NADPH oxidase, an important role in the spatio-temporal regulation of ROS production is also played by enzymes involved in arachidonic acid (AA) metabolism, such as phospholipase A_2_ (PLA_2_), lipooxygenases (LOXs), and cyclooxygenases (COXs), suggesting that a complex regulatory network may take place for proper modulation of redox signaling [16]. 

The NADPH oxidase (NOX) complex was originally identified in phagocytic leukocytes as an enzymatic defense system against infections required for the oxidative burst-dependent microbial killing [17–19]. It is composed of membrane-associated and cytosolic components, which assembly to form the active NOX enzymatic complex in response to appropriate stimuli. Specifically, this complex consists of membrane-associated cytochrome b558, comprising the catalytic gp91^phox^ (also known as NOX2) and regulatory p22^phox^ subunits, and four cytosolic regulatory components, including p40^phox^, p47^phox^, p67^phox^, and the small GTPase Rac1 [17]. The neutrophil expresses two different Rac isoforms, including the phagocyte-specific Rac2 and the more ubiquitously expressed Rac1. Detailed molecular analysis has revealed that Rac proteins function as a necessary switch for ROS generation and that the protein is recruited to the membrane following neutrophil activation where it can bind to both p67^phox^ and gp91^phox^ [20].

Many evidences suggest that certain aspects of neutrophil biology appear to be conserved in the ROS signaling of nonphagocytic cells. In particular, homologues of the NADPH oxidase were found in vascular endothelial cells and smooth muscle cells, as well as in other normal or transformed cells such as colon cancer or melanoma [21]. Several isoforms of the catalytic NOX2 protein were identified, including NOX1, NOX3, NOX4, and NOX5, and shown to localize in proximity of specific redox-sensitive molecular targets within discrete subcellular compartments, thereby facilitating the compartmentalization of redox signaling [15]. In addition, the expression of a constitutively activated form of Rac1 was noted to increase the basal level of hydrogen peroxide in immortalized fibroblasts [22] as well as in certain transformed cell lines [23], while the expression of a dominant negative form of Rac1 was shown to inhibit the production of ROS following addition of various ligands [22]. These data suggest that a Rac-regulated oxidase exists in a wide range of cell types and participates in normal signal transduction.

It has been shown that NOX1 constitutively binds the RacGEF *β*PIX, and the interaction is caused by growth factor stimulation [24]. This and previous studies [25] also support a pathway where ligand addition results in the sequential activation of phosphatidylinositol 3-kinase (PI3K), which in turn generates lipid products that can activate GEFs through the PH (pleckstin homology) domain present within the exchange factors. Activation of the GEF leads to increased Rac activity that is presumed to directly stimulate NOX [14].

The hypothesis that ROS generation is regulated by Rac and the role of ROS as specific effector molecules that act downstream of Rac is supported by several evidences. In a recent paper there has been shown a role of Rac-regulated ROS in the crosstalk between G-protein-coupled receptors (GPCRs) and the JAK/STAT pathway [26], while different studies support a role of Rac1 as a crucial, common upstream mediator of ROS production in integrin-mediated outside-in signaling [11–13, 16, 27].

Several evidences have implicated ROS in the integration of signals from VEGF and Rac to regulate the integrity of the endothelial barrier [22, 28–32]. Further studies demonstrated that the VEGF-dependent phosphorylation of VE-cadherin and *β*-catenin are dependent on Rac and ROS and result in decreased junctional integrity and enhanced vascular permeability [33, 34].

In addition to NADPH oxidase, Rac1 has been demonstrated to act upstream of AA-metabolizing enzymes, such as PLA_2_ [35, 36], 5-LOX [13, 26, 27], and COX-2 [37], whereas many reports show that AA metabolism modulates NADPH oxidase and mitochondrial ROS production [16].

Another aspect of oxidant signaling derived from the initial observation that Rac proteins regulate ROS levels is the demonstration of redox-dependent crosstalk between different small GTPase family members.

ROS production is apparently an essential component in signaling cascades that mediate Rac1/p190RhoGAP-induced downregulation of RhoA and concomitant formation of membrane ruffles and integrin-mediated cell spreading. The pathway linking generation of ROS to downregulation of Rho involves inhibition of the low-molecular-weight protein tyrosine phosphatase (LMW-PTP) and a consequent increase in the activation by phosphorylation of the Rho inhibitor p190Rho-GAP [38]. It has been shown that ROS production causes p190RhoGAP translocation to the adherens junctions (AJs), where it binds p120ctn, and subsequently inhibits local Rho activity [39]. It thus plays a role in the stabilization of cell-cell contacts [34].

These findings suggest that Rac1 downregulates Rho and stress fiber formation in a redox-dependent manner and define a mechanism for the coupling of changes in cellular redox state to the control of actin cytoskeleton rearrangements by Rho GTPases. 

In addition to Rac, the production of ROS by nonphagocytic cell types stimulated by growth factors or cytokines includes the participation of p21Ras [22]. Fibroblasts expressing constitutively active mutants of both Rac and Ras produce high levels of ROS associated with a high rate of proliferation. In the same study experimental evidence was provided suggesting that Rac is positioned downstream to Ras. Similar overexpression of Ras in other cell types such as keratinocytes [40] and epithelial cells [41] also demonstrated an increase in basal ROS levels. The pathway by which Ras regulates the levels of ROS remains incompletely understood. It has been shown that in some cells it proceeds through a PI3K and Rac-dependent pathway [25] leading to the regulation of a NOX-dependent oxidase. In other cell types the source of Ras-induced ROS appears to be linked to the mitochondria [42]. 

Mitochondria have the highest levels of antioxidants in the cell and play an important role in the maintenance of cellular redox status, thereby acting as a ROS and redox sink and limiting NADPH oxidase activity. However, mitochondria are not only a target for ROS produced by NADPH oxidase but also a significant source of ROS, which under certain conditions may stimulate NADPH oxidases. Many findings indicate the existence of a bidirectional signaling crosstalk between mitochondria and NADPH oxidase, where small GTPases can orchestrate a complex web of regulation for ROS production [43–45].

Indeed, in integrin signaling, the regulation of mitochondria by both Rac and RhoA appears to be related to their ability to alter intracellular ROS [12]. 

It has been shown that Nerve Growth Factor- (NGF-) induced differentiation of PC12 cells is mediated by significant alteration of mitochondrial metabolism by reducing mitochondrial-produced ROS and stabilizing the electrochemical gradient. This is accomplished by stimulation of mitochondrial manganese superoxide dismutase (MnSOD) via Ki-Ras and ERK1/2 [46].

Thus ROS produced by small GTPases could regulate mitochondrial properties, including the overall metabolic rate and the generation of mitochondrial oxidants with important signaling functions within the cell [14].

## 3. RasGTPase Regulation by ROS

Although several studies implicate RasGTPases in the production and regulation of intracellular ROS, many evidences indicate that Ras proteins can also be direct targets of ROS. Similar to the action of GEFs, various redox agents, including both ROS and RNS, have been shown to stimulate Ras guanine nucleotide dissociation *in vitro* and upregulate Ras function *in vivo*. 

Lander and coworkers showed for the first time that NO is able to activate Ras by promoting RasGDP dissociation *in vitro*, GTP binding to Ras *in vivo*, and stimulation of pathways downstream to Ras [47–54]. The target site of NO-mediated guanine nucleotide dissociation on Ras is Cys^118^, which is located in the nucleotide-binding NKCD motif [49, 50, 54, 55]. Further studies indicated that •NO_2_, a reaction product of NO with O_2_, reacts with the Ras Cys^118^ thiol to induce a radical-based process leading to stimulation of nucleotide exchange on Ras [56, 57]. In addition to NO, O_2_^•−^ showed to be able to facilitate guanine nucleotide dissociation from Ras as well as the Ras-related GTPase Rap1A. The molecular mechanism of O_2_^•−^-mediated guanine nucleotide dissociation is similar to that of the NO/O_2_-mediated guanine nucleotide dissociation [58]. The redox-sensitive NKCD motif has been found within the Ras subfamily of GTPases such as H, N, K, and E-Ras as well as in Rap1A [7].

Redox-active motifs were afterwards found to be present in other Ras superfamily GTPases, suggesting that redox regulation of GTPase signaling is more widespread that previously envisioned [59].

The GXXXXGK(S/T)C redox-sensitive motif, located in the phosphoryl-binding loop important for redox-mediated regulation of guanine nucleotide exchange activity *in vitro,* was identified and characterized in the Rho family GTPases. This motif contains a redox-sensitive cysteine (Cys^18^, Rac1 numbering) at the C-terminus and it is conserved in almost half of Rho family GTPases such as Rac1 (and its isoforms Rac2 and 3), Cdc42, and RhoA (and its isoforms RhoB and C) [59, 60]. The radical-based molecular mechanism of Rho GTPase guanine nucleotide exchange appears similar in nature to the mechanism characterized for Ras GTPases. 

An *in vivo* study aimed to analyze the effect of exogenous and endogenous ROS on the activation of RhoA in fibroblasts was performed by Aghajanian and coworkers [61]. This study showed that RhoA can be directly activated by ROS in cells by oxidative modification of critical Cys residues within the redox-active motif, and that ROS-mediated activation of RhoA can induce cytoskeletal rearrangement, thus supporting the existence of a novel mechanism of regulating GTPase signaling cascades, independent to classical regulation by GEFs and GAPs, that can affect cytoskeletal dynamics [61].

A number of Rab proteins also have the GXXXXGK(S/T)C motif (Rab1B, Rab2A/B, Rab4A/B, Rab14, Rab15, Rab19, and Sec4). Intriguingly, many Rab GTPases (Rab1A, Rab8A/B, Rab10, and RAb13) possess both the NKCD and GXXXXGK(S/T)C motifs, whereas some Rab proteins (Rab3A/B/C/D, Rab 7, Rab22, and Rab38) possess only the NKCD motif [7]. 

A CGNKXD redox-sensitive motif was found in Ran protein [62]; this motif contains a redox-sensitive cysteine, Cys^120^, at the N-terminal. In addition to this CGNKXD motif, Ran possesses an additional redox-sensitive cysteine Cys^85^ (Ran numbering). This type of redox center is also conserved in Dexras1 and Rhe proteins as well as in some Rab GTPases [62].

Although redox regulation of the members of Rab and Ran families has been recently discovered, its physiological relevance and pathological consequences linked to the misregulation of redox signaling associated with these redox sensitive small GTPases have not yet been explored [7]. 

## 4. Pathological Implications of the Interplay between Small GTPases and ROS

Over the past several years, it has become clear that ROSs play an important role in physiological processes like cell differentiation, proliferation, migration, and vasodilatation. On the other hand, production of ROS “in the wrong place at the wrong time” results in oxidative stress leading to cellular dysfunction and apoptosis, which contributes to different pathologies like atherosclerosis, heart failure, hypertension, ischemia/reperfusion injury, cancer, aging, and neurodegeneration [40].

There is a vast body of literature that links vascular ROS production to cardiovascular disease [63]. Vascular ROS production as well as Rac1 activation has been associated with hypertrophy and smooth muscle cell proliferation, endothelial dysfunction as well as endothelial cell migration, hypertension inflammation, and atherosclerosis [64–67]. Vascular hypertrophy has been ascribed to the effects of various receptor agonists, including Angiotensin II (Ang II), which induces ROS production in VSMCs in a Rac1-dependent fashion [68]. Recent studies showed that this Ang II-induced ROS production also requires the membrane adapter caveolin, which is involved in Rac1 activation [69, 70], and the lipid kinase PI3K-*γ* [68, 71].

Ischemia/reperfusion (I/R) injury is also associated with ROS production. This is a clinically relevant problem occurring as damage to the myocardium following blood restoration after a critical period of coronary occlusion. It is well known that immediately following the reinstitution of oxygenated blood into ischemic tissue, there is a rapid burst of ROS, but the molecular basis and source of this process are not yet convincingly identified [14, 72]. However, both *in vitro* and *in vivo* experiments [73, 74] have suggested that Rac1 plays a dominant role in ROS generation after I/R, and it activates the nuclear factor NF-*κ*B and stimulates mRNA expression of several inflammatory genes, such as TNF-*α* and iNOS in the liver, leading to massive hepatocyte necrosis. Thus, efforts aimed at inhibiting Rac protein function could be useful therapeutic strategies in a variety of clinical settings in which there is concern about the potential harmful effects of I/R injury [73, 74].

Data from the literature suggest that ROS and RhoA activation are associated to airway smooth muscle contractility [75–77]; it has been shown that oxidative stress with H_2_O_2_ leads to airway smooth muscle contraction mediated by increases in intracellular Ca^2+^ concentration and the Rho/Rho kinase pathway [77].

Both ROS and Rho/Rho kinase have been suggested to play important roles in vasoconstriction and may contribute to the pathogenesis of hypertension in experimental animals and humans. Jin and coworkers demonstrated the direct activation of the Rho/Rho kinase signaling pathway by ROS in rat aorta, suggesting an important role for ROS-mediated Rho/Rho kinase activation in vasoconstriction [78].

As previously discussed, Aghajanian and coworkers proposed a novel mechanism for the regulation of RhoA in cells by ROS that allows predicting that ROS may directly activate Rho signaling in smooth muscle and in the endothelium thus affecting vascular permeability. This mechanism of regulation, which is independent of classical regulatory proteins, may be particularly relevant in pathological conditions where ROSs are generated and the cellular redox-balance altered, such as in asthma and I/R injury [62].

It is well known that activated Ras signaling contributes to oncogenic transformation by providing molecular signals that promote cell proliferation, obstruct cell death, inhibit cellular differentiation, and induce angiogenesis [79]. Signaling pathways starting from activated Ras and resulting in mitochondrial ROS production and downstream signaling regulation have been the subject of several recent interesting studies, and different mechanisms have been proposed to elucidate the role of mitochondrial respiration in cancer. 

It has been shown that the activation of K-Ras(G12V) causes modifications in mitochondrial metabolism finalized to support growth under hypoxic conditions, and leading to increased generation of ROS [80]. The major source of ROS generation required for KRas-induced anchorage-independent growth is the Q_o_ site of mitochondrial complex III [81]. Thus mitochondrial dysfunction appears to be an important mechanism by which K-Ras(G12V) causes metabolic changes and ROS stress in cancer cells and promotes tumor development [80]. 

Mitochondrial dysfunction and ROS production mediated by activation of Ras, Myc, and p53 produce downstream signaling (e.g., NF*κ*B, STAT3, etc.) that are crucial in cancer-related inflammation. Different inflammation-associated cancers resulting from signaling pathways coordinated at the mitochondrial level have been identified that may prove useful for developing innovative strategies for both cancer prevention and cancer treatment [82].

Several studies suggest that autophagy may be important in the regulation of cancer development and progression and in determining the response of tumor cells to anticancer therapy [83]. A recent paper shows that autophagy is associated with the malignant transformation of mammalian cells induced by K-Ras and that ROSs are involved as signaling molecules in K-Ras(G12V)-induced autophagy. The increase in intracellular ROS produced in response to oncogenic K-Ras involves p38 MAPK signaling and leads to JNK activation. JNK acts downstream of ROS and plays a causal role in autophagy induction through upregulation of autophagy-specific genes 5 and 7 (ATG5 and ATG7) [84]. As mitochondria sustain viability of Ras-expressing cells in starvation, autophagy is required to maintain the pool of functional mitochondria necessary to support growth of Ras-driven tumors [85]. These findings provide new insights into the relationship between autophagy and oncogenesis and suggest that targeting autophagy and mitochondrial metabolism are valuable new approaches to treat cancers with Ras mutations.

Oncogenic activation of the *H-Ras* gene has been found in more than 35% of patients with urothelial carcinomas [86]. It has been recently shown that in addition to tumorigenic ability, oncogenic H-Ras possesses a novel proapoptotic activity to facilitate the induction of apoptosis by histone deacetilase inhibitors (HDACIs), a new class of anticancer agents characterized by high cytotoxicity toward transformed cells [87]. Expression of oncogenic H-Ras in human bladder tumor J82 cells and treatment of cells with the HDACI, FK228, sinergistically induce the ERK pathway, resulting in differentially increased NOX-1 elevation and ROS production, leading to differential activation of caspases and cell death [88–90]. Thus, in addition to its well-known role in mediating mitogenic signals for cell proliferation and transformation, the ERK pathway plays an essential role in mediating apoptotic signals induced by HDACIs through induction of NOX-1 elevation to ROS production and caspase activation for inducing cell death. In addition, expression of oncogenic H-Ras in J82 cells also results in an increased susceptibility to exogenous H_2_O_2_ for inducing caspase activation and apoptosis [88]. Further studies revealed that FK228 combined with exogenous H_2_O_2_ cooperatively induces activation of MEK1/2 and ERK1/2 to increase NOX-1 elevation, intracellular ROS production, caspase activation, and cell death. Expression of oncogenic H-Ras significantly increases these FK288- and exogenous H_2_O_2_-induced effects. Oncogenic H-Ras-increased susceptibility to FK228 could be alternatively achieved by additional treatment with exogenous H_2_O_2_. These findings have important and useful implications as combined use of HDACIs with ROS-generating agents may apply to therapeutic strategies to preferentially kill malignant cells with or without oncogenic H-Ras activation [91].

Due to the crucial role played by Ras in many cellular signaling cascades, diseases relevant to dysregulation of redox signaling often result in deregulation of Ras-dependent cellular signaling events. Since the first identification of the redox-sensitive NKCD motif of Ras [51], considerable pathophysiological data are available, including some bearing directly on the relevance of redox-mediated misregulation of the Ras NKCD motif to certain diseases [7]. Rap1A, another reprehensive protein that possesses the NKCD-motif, is a regulator of NAD(P)H oxidase. However, a pathophysiological outcome associated with the misregulation of Rap1A redox signaling has not been clearly investigated [7].

Cancer is one of the most prevalent disorders caused by misregulation of Ras activity by a redox agent. Numerous studies show that cancers, to a large extent, are induced by misregulation of Ras redox signaling combined with an alteration of Ras downstream cellular transduction cascades. As with cancers, many cardiovascular and neuronal disorders appear to be the result of dysregulation of various cellular signaling events via the redox-sensitive Ras (for a deeper investigation see [7]).

The misregulation of the redox signaling of Ras with its downstream cascades also has been linked to various disorders linked with immune and embryo developments. The Ras-dependent activation of Raf also leads to stimulation of a phosphorylation of Ets-like protein-1 and tumor necrosis factor-*α* messenger RNA induction; both actions suggest that NO, through the Ras-dependent Raf-MEK1/2-ERK1/2 pathway, modulates a host's defenses and the inflammation of T lymphocytes [92]. ROS-mediated signaling via Ras, NF-*κ*B, and related transducers may link to embryopathies [93].

## 5. Concluding Remarks

Although, for many years, the generation of intracellular redox agents was viewed solely as the unregulated by-product of aerobic metabolism and other enzymatic processes, over the last years a growing number of reports have suggested that the production of intracellular ROS is tightly regulated and that these redox agents serve as intracellular signaling molecules being involved in a variety of cell signaling pathways. Here we have reviewed studies reporting that members of the RasGTPase superfamily are able to regulate intracellular ROS production, and that the production of ROS by small GTPases is an important aspect of the function of these monomeric G-proteins. In addition, the functional cross-talk between some different RasGTPase family members (see Rac1 and RhoA) appears strictly related to redox signaling. Finally, due to the presence of conserved redox-sensitive sequences, many of the Ras superfamily small GTPases have been shown to be targets of ROS regulation. 

Thus, redox agents, as upstream regulators and/or downstream effectors of redox-sensitive RasGTPases, strongly contribute to their overall biological function playing a key role in various cellular signaling events. Dysregulation of small GTPases by redox agents or dysregulation of redox signaling by small GTPases may significantly alter cellular signaling pathways and lead to the pathological state. 

Given the prominent role the RasGTPase family members play in regulating fundamental cell processes like growth, migration, adhesion, cytoskeletal integrity, survival, and differentiation, the comprehension of molecular mechanisms of the interplay between small GTPases and ROS may strongly help to clarify how redox agents contribute to physiological and pathological cellular events and provide novel strategies for treatment of many pathological conditions where both RasGTPases and oxidative stress play a role.

## Figures and Tables

**Table 1:** The Ras superfamily of small GTPases. The RasGTPase superfamily is divided into 9 families of small GTP-binding proteins on the basis of sequence and functional similarities (modified from [7]).

Ras	Rab	Rho	Sec	Arf	Rad	Ran	RheS	Rit
H-Ras	Rab1A	RhoA	N-Sec1	Arf1	Rad	Ran/TC4	Rhes	Rit
K-Ras	Rab1B	RhoB	S-Sec1	Arf2	Gem	Dexras1	Others	Rin
N-Ras	Rab2	RhoC	Sec4	Arf3	Kir	Others		Ric
E-Ras	Rab3A	RhoD	Sly1p	Arf4	Rem1			Others
TC21	Rab3B	RhoE	Others	Arf5	Rem2			
R-Ras	Rab4	Rnd1		Arf6	Others			
M-Ras	Rab5A	Rnd2		Arf7				
Rap1A	Rab5B	RhoG		Others				
Rap1B	Rab6	Rac1						
Rap2A	Rab7	Rac2						
Rap2B	Rab8	Rac3						
RalA	Rab9	Cdc42						
RalB	Rab10	TC10						
Others	Others	TTF						
		Others						
